# Evaluation of variable selection methods for random forests and omics data sets

**DOI:** 10.1093/bib/bbx124

**Published:** 2017-10-16

**Authors:** Frauke Degenhardt, Stephan Seifert, Silke Szymczak

**Affiliations:** 1 Institute of Clinical Molecular Biology, Kiel University, Germany; 2 Institute of Medical Informatics and Statistics, Kiel University, Germany

**Keywords:** machine learning, random forest, feature selection, high dimensional data, relevant variables

## Abstract

Machine learning methods and in particular random forests are promising approaches for prediction based on high dimensional omics data sets. They provide variable importance measures to rank predictors according to their predictive power. If building a prediction model is the main goal of a study, often a minimal set of variables with good prediction performance is selected. However, if the objective is the identification of involved variables to find active networks and pathways, approaches that aim to select all relevant variables should be preferred. We evaluated several variable selection procedures based on simulated data as well as publicly available experimental methylation and gene expression data. Our comparison included the Boruta algorithm, the Vita method, recurrent relative variable importance, a permutation approach and its parametric variant (Altmann) as well as recursive feature elimination (RFE).

In our simulation studies, Boruta was the most powerful approach, followed closely by the Vita method. Both approaches demonstrated similar stability in variable selection, while Vita was the most robust approach under a pure null model without any predictor variables related to the outcome. In the analysis of the different experimental data sets, Vita demonstrated slightly better stability in variable selection and was less computationally intensive than Boruta.

In conclusion, we recommend the Boruta and Vita approaches for the analysis of high-dimensional data sets. Vita is considerably faster than Boruta and thus more suitable for large data sets, but only Boruta can also be applied in low-dimensional settings.

## Introduction

Owing to recent technological advances it is possible nowadays to characterize patients or healthy controls at multiple omics levels. For example, expression of >20 000 mRNA transcripts or the methylation status at >400 000 CpG sites in the genome can be measured using microarrays. Next-generation sequencing (NGS) technologies enable even larger numbers of molecules to be quantified. Although different technologies are used for different omics levels, the resulting data sets have several common characteristics making their analysis challenging. The number of variables is often much larger than the number of individuals. Furthermore, the data sets are usually sparse regarding relevant information, i.e. only a small set of variables is associated with the outcome. Additionally, complex correlation patterns are present between the variables.

Machine learning methods are promising computational approaches for classification and regression utilizing high-dimensional omics data sets. A particularly well-suited method to tackle the presented challenges is random forest (RF) [1], an ensemble learning method based on decision trees. RF has been successfully applied in genetic [2], gene expression [3], methylation [4], proteomics [5] and metabolomics studies [6]. It is a flexible approach that can be used to both perform classification, i.e. predicting case-control status, and regression, i.e. predicting quantitative traits.

In many applications a well-performing prediction model is only one of the goals. Another, sometimes even more important, aim is to identify those variables that enable this good prediction, i.e. to reduce the large set of measured variables to the ones that contain more information than noise. RFs provide variable importance measures, which can be used to rank variables based on their predictive importance. However, it is difficult to distinguish relevant from irrelevant variables based on this ranking only. Therefore, several variable selection procedures have been proposed that use different criteria and approaches to report the set of truly relevant variables.

One popular approach that is also used in combination with other machine learning methods is recursive feature elimination (RFE) [7]. RFE uses the prediction error to select a minimal set of variables needed for a good prediction. Hence, only a limited number of variables need to be measured for further application of the prediction model. However, for many clinically relevant outcomes such as survival or response to treatment, different sets of variables with only few or even no common genes might lead to similar prediction performances [8, 9]. These sets might come from independent studies or are defined based on slightly different versions of the training data that are generated by bootstrapping or subsampling. The main reason for this finding is that many omics variables are correlated and therefore carry redundant information regarding prediction [10]. Including these redundant variables in the variable set will lead to more consistent results and provide a more complete picture of the pathological mechanisms driving disease or prognosis [9, 11]. In addition to the improved interpretability, prediction models based on larger sets of variables might have better prediction performance on independent data sets [12].

Evaluating prediction performance to determine important variables is suitable for approaches that aim to extract a minimal set of discriminative predictor variables like RFE. In contrast, alternative variable selection methods that do not remove redundant variables, estimate the distribution of importance values of irrelevant variables and select predictor variables with significantly larger importance values. These approaches are becoming more popular [13, 14] and our comparison study includes not only well-known methods such as permutation (Perm), its parametric variant (Altmann) [15] and the Boruta algorithm [16], but also recently published approaches like recurrent relative variable importance (r2VIM) [17] and the Vita method [18].

We perform a systematic comparison study using both simulated and experimental data. The two simulation setups contain correlated predictor variables. While the first study is based on simple correlation structures, more complex and realistic correlation patterns are estimated from experimental data and used in the second study. We also analyze experimental methylation and gene expression data sets using data from two independent studies for each classification setting. In each of the studies, prediction performance and stability of variable selection are assessed. In the simulation studies we additionally evaluate sensitivity, empirical power and false discovery rate (FDR; only Simulation Study 1).

In the next section, we first explain the RF approach before we describe the different variable selection methods in more detail. We then present the simulation studies and the experimental data sets, followed by results and discussion.

## Random forest

RF is an ensemble learning method based on classification and regression trees [1]. Each tree is trained on a bootstrap sample, and optimal variables at each split are identified from a random subset of all variables. The selecting criteria are different for classification and regression problems. For the former setting, the Gini index is applied, whereas variance reduction is used for the latter approach. The global prediction of the RF is calculated as a majority vote or average for classification or regression, respectively. Regression mode can also be used to obtain a binary outcome enabling estimation of probabilities similar to logistic regression [19].

In addition to prediction, RFs can be used to estimate variable importance measures to rank variables by predictive importance. One popular variable importance is the mean decrease of the Gini index. However, it has been shown that this method is biased [20] and therefore it is not included in our comparison study. Instead, the permutation importance of a variable is used, which is calculated as the difference of prediction performance before and after permuting the values of the variable averaged over all trees. In each tree, only out-of-bag observations, i.e. observations that were not used for training the forest, are included in the importance calculation. Variables that are relevant for prediction will have large importance values, whereas variables that are not associated with the outcome have values close to zero.

## Variable selection methods

Different approaches have been proposed to identify the most important variables based on the ranking mentioned in the previous subsection. An overview of the characteristics and original implementations of the methods that are compared in this study is provided in Table 1.

**Table 1:** Information about the different variable selection approaches that are compared

Abbreviation	Name	Goal	Approach	R package	Ref.	**Citations** ^a^
Altmann	Altmann	All relevant variables	Permutation of outcome; parametric *P*-value	R code on first author’s Web site (http://www.altmann.eu/documents/PIMP.R) Implemented in ranger package (https://cran.r-project.org/web/packages/ranger/index.html) and vita package (https://cran.r-project.org/web/packages/vita/index.html)	[15]	40
Boruta	Boruta	All relevant variables	Importance significantly larger than those of shadow variables	Boruta (https://cran.r-project.org/web/packages/Boruta/index.html)	[16]	103
Perm	Permutation	All relevant variables	Permutations of outcome; nonparametric *P*-value	No specific implementation for RF	–	–
r2VIM	Recurrent relative variable importance	All relevant variables	Relative importance based on minimal observed importance; several runs of RF	r2VIM (http://research.nhgri.nih.gov/software/r2VIM)	[17]	2
RFE	Recursive feature elimination	Minimal set	RF with smallest error based on iterative removal of least important variables	varSelRF (https://cran.r-project.org/web/packages/varSelRF/index.html)	[7]	671
Vita	Vita	All relevant variables	*P*-values based on empirical null distribution based on non-positive importance scores calculated using hold-out approach	Vita (https://cran.r-project.org/web/packages/vita/index.html) Implemented in ranger package (https://cran.r-project.org/web/packages/ranger/index.html)	[18]	0

^a^Based on Web of Science (24 July 2017).

For better comparability in our study we reimplemented several of the approaches in a common framework that is based on the ranger R package [21]. This new implementation of RFs is more time and memory efficient compared with the standard RF implementation in the randomForest R package. Therefore it is better suited for large data sets that are common in omics studies. Our reimplementations are provided as an R package called Pomona (the Roman goddess of fruits) on GitHub (https://github.com/silkeszy/Pomona).

Another alternative to the variable selection approaches included in our study is a method called VSURF [22], which is implemented in an R package with the same name [23]. However, the method seems to be sensitive to the parameter settings and it was not possible to analyze the experimental data sets in a reasonable amount of time (data not shown).

### Recursive feature elimination

RFE aims to find a minimal set of variables, which leads to a good prediction model [7]. It starts with a RF built on all variables. A specific proportion of the least important variables is then removed and a new RF is generated using the remaining variables. These steps are recursively applied until a single variable is left as input. At each step the prediction performance is estimated based on the out-of-bag samples that were not used for model building. The set of variables that leads to the RF with the smallest error or to an error within a small range of the minimum is selected. The original method as implemented in the R package varSelRF calculates variable importance only once based on the RF with all variables. However, we recompute the rankings at each step because it has been shown that this modified algorithm is more efficient in case of correlated predictors [24].

RFE is a popular method for variable selection and it has been cited >500 times in Web of Science. It is often applied to analyze high-dimensional molecular data sets generated, e.g. in transcriptomics [25], proteomics [26] and metabolomics [27] experiments.

### Boruta

The Boruta algorithm is named after a god of the forest in the Slavic mythology and was developed to identify all relevant variables within a classification framework [16]. The main idea of this approach is to compare the importance of the real predictor variables with those of random so-called shadow variables using statistical testing and several runs of RFs. In each run, the set of predictor variables is doubled by adding a copy of each variable. The values of those shadow variables are generated by permuting the original values across observations and therefore destroying the relationship with the outcome. A RF is trained on the extended data set and the variable importance values are collected. For each real variable a statistical test is performed comparing its importance with the maximum value of all the shadow variables. Variables with significantly larger or smaller importance values are declared as important or unimportant, respectively. All unimportant variables and shadow variables are removed and the previous steps are repeated until all variables are classified or a pre-specified number of runs has been performed.

The original implementation based on the standard randomForest R package [28] was computationally intensive, which made its application in large omics data sets challenging. Nevertheless, the Boruta approach has been used in >100 studies, including omics data sets resulting from gene expression [29] and microbiome data analysis [30]. In the 5.0 version of the Boruta package, the ranger package is used for RF training and variable importance estimation.

### Permutation approach

In contrast to the Boruta approach, where values of the predictor variables are permuted, a standard permutation test can be applied to estimate the distribution of importance scores under the null hypothesis of no association between predictor variables and outcome. The Perm approach therefore permutes values of the outcome, which leaves correlation patterns between predictor variables untouched. In our comparison, outcome permutations are repeated several times, and in each step, a RF is trained and variable importance values under the complete null hypothesis are estimated. Predictor variables with original importance values larger than importance values in all permutation runs are selected as important.

### Altmann

Perm determines empirical (nonparametric) *P*-values based on the null distribution of variable importance scores after permuting the outcome. To reduce the number of permutations, Altmann *et al.* [15] proposed to use parametric *P*-values by fitting a defined probability distribution such as normal, lognormal or Gamma to the empirical distribution of null importance values. Parameters of these distributions are estimated using maximum likelihood methods and *P*-values are calculated as the probability of observing an importance score that is larger than the original importance score under the estimated distribution.

In our comparison we used the normal distribution, which has been shown to work well in practice, see e.g. [18]. However, we would like to note that a different distribution might be more appropriate if a different variable importance score is used. For example, a Z score defined on a scaled version of the permutation importance is not normally distributed [31]. Furthermore, a statistical test based on these values has undesirable properties [32]. Although the Altmann approach was published in the same year as the Boruta approach, it has only been cited 40 times but used in several omics studies, e.g. analyzing gene expression [33] and microbiome data [34].

### Recurrent relative variable importance

The idea of r2VIM is based on the assumption that omics data sets usually contain many unimportant variables, which can be used to estimate importance values of null variables [17]. Several RFs are generated based on the same data set and parameter values differing only in the seed of the random number generating process. Each RF is used to calculate importance values, which are divided by the absolute minimal importance value observed in each run resulting in relative values that can be interpreted more easily. In case the minimal observed importance is exactly zero in a specific RF run, the minimal importance value over all runs is used as denominator. Variables with a minimal relative importance value greater or equal to a specified factor are selected and defined as important variables.

To identify genetic variants that are associated with complex diseases, r2VIM has been developed [17]. In simulated genome-wide association data sets, it controlled the number of false positives if a factor of 3 or larger is used. Compared with standard statistical methods, such as logistic regression, the power to detect causal variants is only slightly decreased. The method can also be applied to identify gene–gene interactions [35].

### Vita

The Vita algorithm proposed by Janitza *et al.* [18] is similar to r2VIM, as it uses only the existing data without any permutations to estimate the null distribution of variable importance scores. The observed non-positive variable importance scores are used to construct a distribution that is symmetric around zero. The authors showed that variable importance values of null variables that are calculated using out-of-bag samples are positively skewed and a symmetric distribution is only achieved using a special cross-validation procedure (called hold-out approach). The overall data set is divided into two equally sized subsets, and two RFs are trained using either one of the sets. Variable importance is then estimated based on the other, independent set. The final importance values, called hold-out importance, are calculated by averaging the two estimated scores per variable. Based on the resulting empirical distribution, *P*-values can be calculated.

Because this approach has been published recently, no studies citing the method could be identified.

## Performance comparison

Because both experimental and simulated omics data provide specific advantages to compare the different variable selection methods, we used both types of data sets for our comparison. In simulation studies the true substructure in the data is known, and the application of statistical methods enables the estimation of power and FDRs. However, real experimental omics data sets feature complex properties that are difficult to simulate, e.g. usually they have complex correlation structures. Hence, investigation of experimental data is obligatory to obtain reliable results to evaluate statistical and computational methods under realistic analysis settings.

In this examination we included two different simulation studies: In the first one the relationship between the quantitative outcome and a small number of predictor variables is nonlinear and therefore well suited for an analysis using the RF approach. In contrast, correlation between predictor variables is defined in a simple block structure. In addition, the data sets contain many uncorrelated variables that are independent of the outcome to analyze the risk of false-positive findings. In contrast, the second simulation study is based on a classification setting and more closely resembles omics data because correlation patterns estimated from experimental data were simulated. Because all of the predictor variables were strongly correlated with at least one of the other variables, it is not possible to evaluate false positives because selected variables are either directly influencing the outcome or are correlated with at least one of those variables.

### Simulation Study 1

#### Data

For the simulated data in this study we used a modified version of a nonlinear regression model with correlated predictor variables, which has been used previously [36]. The quantitative outcome depends on three variables according to the formula

$$y=0.25\;\text{exp}\;\;(4x_{1})+\frac{4}{1+\text{exp}\;\left(-20\left(x_{2}-0.5\right)\right)}+3x_{3}+\varepsilon$$

Where ε was normally distributed with mean 0 and standard deviation 0.2 $\left(N\left(0,0.2\right)\right)$. The three variables $x_{1}$, $x_{2}$ and $x_{3}$ as well as three additional variables $x_{4}$, $x_{5}$ and $x_{6}$ were independently sampled from a uniform distribution on the interval 0 and 1 ($U\left(0,1\right)$) and used as basis for generating the correlated predictor variables. They were simulated according to

$$v_{i}^{\left(j\right)}=x_{i}+\left(0.01+\frac{0.5\left(j-1\right)}{n-1}\right)\cdot N\left(0,0.3\right)$$

for j=1,…,n and i=1,…, 6, where $v_{i}^{\left(j\right)}$ denotes the j-th variable in group i and n is the size of each group. Note that correlation between the base variable $x_{i}$ and $v_{i}^{\left(j\right)}$ decreases as j increases. Furthermore, additional predictor variables that are uncorrelated with any of the base variables and each other were simulated based on a uniform distribution $U\left(0,1\right)$.

We considered four different simulation scenarios: The first two included the causal variables $v_{i}^{\left(j\right)},i=1,2,3$ as well as the correlated, non-causal variables $v_{i}^{\left(j\right)},i=4,5,6$ and differed in group size n, for which we used the values 10 and 50. The two other scenarios use the same group sizes and are null models, i.e. the outcome does not depend on any of the simulated predictor variables. Here, variables $x_{i},i=1,2,3$ were only used to simulate correlated variables $v_{i}^{\left(j\right)}$. The outcome was simulated based on three independent, normally distributed variables ($N\left(0,0.2\right)$).

For each of the four scenarios, 100 replicates with 100 individuals and a total number of 5000 predictor variables were simulated. The first 50 replicates were used for variable selection and training a final model, whereas the remaining 50 replicates were used for estimating prediction performance (see below). Parameters for RF and each method can be found in Table 2. Method-specific parameters were selected based on the analysis of the first simulation scenario so that the different approaches lead to similar FDRs (data not shown).

**Table 2:** Parameters used for RF and variable selection methods

Approach	Parameter	Description	Value
RF	ntree	Number of trees	10 000
	mtry	Number of variables selected at each split	33% of number of variables
	nodesize	Minimal number of individuals in terminal node	10% of sample size
Altmann	no.perm	Number of permutations	50
	p.t	Threshold for *P*-values	0
Boruta^a^	pValue	Confidence level	0.01
Perm	no.perm	Number of permutations	500
	p.t	Threshold for *P*-values	0
r2VIM	no.runs	Number of RFs to be generated	20
	factor	Minimal relative importance score for a variable to be selected	3
RFE	prop.rm	Proportion of variables removed at each step	0.1
	tol	Acceptable difference in optimal performance (in %)	10
Vita	p.t	Threshold for *P*-values	0

^a^Using default parameters (mtry = square root of number of variables; nodesize = 1 for classification, 5 for regression).

#### Evaluation criteria

We used the parameters FDR, sensitivity, empirical power, stability and root mean squared error (RMSE) to evaluate the performance of the different variable selection approaches. A false-positive finding means the selection of any predictor variable except the causal variables $v_{i}^{\left(j\right)},i=1,2,3$, by the variable selection method. FDR is defined as the frequency of false-positive findings among all variables selected per method and replicate. Sensitivity is defined as the proportion of correctly identified causal variables among all causal variables on a single replicate. The empirical power of each causal variable is calculated as the frequency of correct selections among all replicates. To estimate stability of variable selection, all pairwise combinations of replicates are considered. For each pair, the stability of the two lists of selected variables by the two replicates was determined using the Jaccard’s index, which is calculated as the division of the length of the intersection and the length of the union of the two sets of variables [10]. This index is 0 if the two sets do not overlap and 1 if the two sets contain the same variables. The average of all pairs is used as stability value for the particular method [37]. Note that stability in our setting is evaluated across independent replicates simulated under the same model, in contrast to other studies where stability is assessed regarding small changes in the original data set such as drawing bootstrap samples [10]. To evaluate prediction performance, a RF using only the selected variables is trained on the same replicate used for variable selection. The corresponding RMSE is then estimated using an independent replicate simulated only for this particular purpose.

### Simulation Study 2

#### Data

For this simulation strategy the correlation structure of the predictor variables was obtained from experimental data as reported previously [18]. Here we estimated the covariance matrix of a gene expression data set from breast cancer patients containing 12 592 genes (see ‘Experimental data sets’ section for details). Gene expression data for 200 individuals were simulated by assuming a multivariate normal distribution with a mean vector of zeros and the estimated covariance matrix using the R package Umpire [38]. Subsequently, 200 causal variables were selected by randomly choosing 25 variables for every effect size from the set {−3, −2, −1, −0.5, 0.5, 1, 2, 3}. To create a binary outcome, the means of the chosen causal variables were changed according to the corresponding effect size in 100 of the 200 individuals. We simulated two pairs of replicates with identical causal variables and effect sizes. For each pair, each replicate was in turn used to select variables and to train a final model as well as to estimate prediction performance. We simulated 50 pairs of replicates resulting in 100 replicates in total, but we used only 20 pairs for the time-consuming approach Perm.

#### Evaluation criteria

We used the parameters empirical power, stability and classification error to evaluate the results of this simulation study. Empirical power was calculated separately for each absolute effect size as the frequency of correctly selected causal variables among all selected variables. Stability was determined using Jaccard’s index for each pair of replicates as described in Simulation Study 1 (see above). Classification error for each replicate in a pair was calculated based on the RF trained on the other replicate, and the mean error was reported for each pair.

### Experimental data sets

#### Data

For the investigation of experimental data, we focused on biological questions for which two large data sets with different individuals are available in public repositories. Using two data sets has several advantages. Stability of selected variables across the two data sets can be estimated and RF models based on selected variables can be trained on one of the data sets and tested on the other. Furthermore, it is not required to divide the data sets for this evaluation, which would decrease the number of individuals and therefore the power to identify biologically relevant variables. An overview of the selected studies and their characteristics is given in Table 3.

**Table 3:** Overview of the experimental studies used for the comparison of variable selection approaches

Name	Data type	Number of variables	Study	Outcome	
Sex	Methylation	21 761 CpG positions		Female	Male
			Adkins	66	74
			Mozhui	109	105
Breast cancer	Gene expression	12 592 genes		ER neg^a^	ER pos^b^
			TCGA (array)	117	396
			TCGA (RNA-Seq^c^)	120	398

^a^Estrogen receptor-negative breast cancer.

^b^Estrogen receptor-positive breast cancer.

^c^RNA-sequencing.

The first research question deals with sex classification based on methylation data. With the different sex chromosomes in males and females as well as X chromosome inactivation in females, most of the CpG positions on the X chromosome should show methylation levels around 0.5 in females, whereas the values in males should be close to either 0 or 1. We used two studies that measure methylation levels using the Illumina 27k array in cord blood of newborns [39, 40]. Preprocessed beta values were downloaded from the NCBI GEO database [41] (accession numbers: GSE27317 and GSE64940). Probes that did not map uniquely to the human genome [42], contained either a common single nucleotide polymorphism in their probe sequence [42] or missing values were removed as well as individuals for which reported sex did not match sex estimated based on median methylation values for probes on the X chromosome. We restricted our analyses to the 21 761 CpG positions available in both data sets of which 815 are on the X chromosome outside of the pseudo-autosomal regions. For comparability, in each of the two studies, methylation values of each CpG position were divided into four different groups based on the quartiles and these ordinal values were used as predictor variables.

The second research question is prediction of estrogen receptor status in breast cancer patients using gene expression data. We created two different data sets based on the technology that was used to measure gene expression in patients available in The Cancer Genome Atlas (TCGA) [43]. For about half of the patients, measurements based on a custom Agilent microarray were available, and for the remaining patients we used NGS-based expression data. Clinical information and normalized expression measurements of primary tumor samples were downloaded from GDAC Firehose (http://gdac.broadinstitute.org/) [44]. Genes with missing values (array) or zero counts in >10% of the samples (NGS) or multiple locations in the genome were removed. Read counts were log transformed (*log_2_(x + 1)*). Technology-specific identifiers were converted to Ensembl gene identifiers using the Bioconductor package AnnotationHub version 2.4.2 (Ensembl GRCh37.75). To enable a comparison between the two technologies, expression values of each gene were standardized to a mean of 0 and standard deviation of 1 separately in the two data sets. In total, 12 592 genes were available in both data sets and used in the analyses.

#### Evaluation criteria

For both research questions, variable selection using the different approaches was performed on each data set with each method, and a RF was trained with only the previously selected variables as predictor variables. The classification error of the other data set was then used to evaluate prediction performance. In addition, the stability of variable selection was assessed by comparing the two variable lists generated on the two data sets using the Jaccard’s index (see description in simulation study) [10]. For the breast cancer data, we additionally report a modified version of the Jaccard’s index. Owing to technological differences more genes were identified in the RNASeq data so that we used the minimum number of selected genes in the two data sets as denominator of the index. We used the same parameters for RF training and variable selection as in the simulation study (Table 2).

The only difference between the analyses of simulated and experimental data was the mtry value in sex classification data sets, where we had to reduce it to the default value (= square root of number of variables) to estimate the distribution of null predictor variables in the r2VIM method. For the evaluation of run time, we used a single compute node of a computer cluster with 16 CPUs and 40 GB RAM.

## Results

### Simulation Study 1

**Figure 1:**
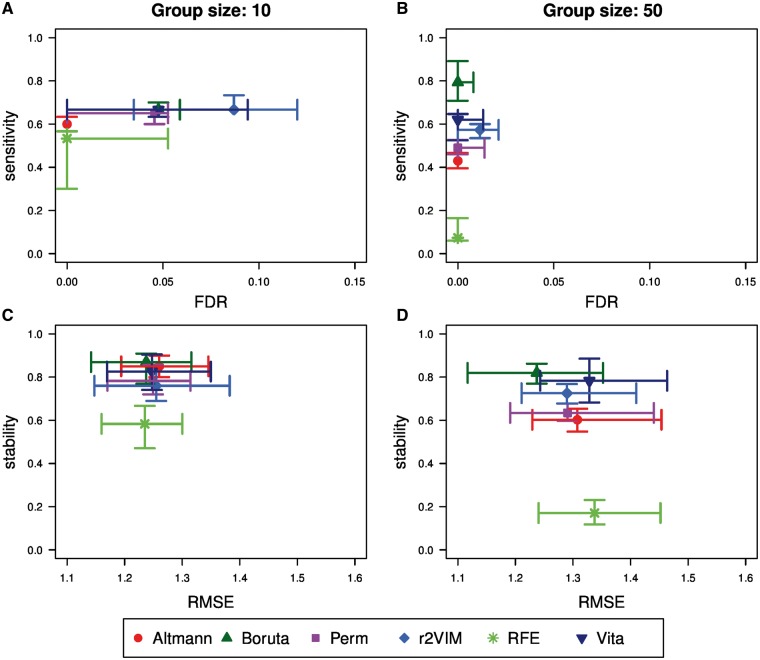
Performance comparison in Simulation Study 1 based on simulations with true effects. Shown are FDRs versus sensitivity of the scenarios with a group size of 10 (**A**) and 50 (**B**) as well as RMSE versus stability for the scenarios with a group size of 10 (**C**) and 50 (**D**). Each subfigure displays the median as well as the interquartile range over all 50 replicates of each method using different plotting symbols and colors.

Results for the two simulation scenarios with true effects in Simulation Study 1 are summarized in Figure 1.

Each of the subfigures shows two evaluation criteria, and an optimal method would be located in the upper left corner, i.e. would have high sensitivity and low FDR. As expected, FDRs of the different methods are generally low; however, they are larger in the scenario with the smaller group size of 10, i.e. with only few causal variables. Only RFE and Altmann have a median FDR of zero in this setting, whereas r2VIM has a median FDR of nearly 0.09. In addition, this approach is the only one with a median FDR greater than zero in the simulation scenario with group size 50. Most of the false-positive variables were selected in a single replicate, while three variables were chosen in two replicates. The median sensitivity is about 60% for all methods in the scenario with group size 10. However, it is more variable in the scenarios with group size 50, i.e. 150 correlated causal variables. The most sensitive approaches are Boruta with a sensitivity of 79% and Vita with 62%, while the sensitivity of RFE is only 7%. The RMSE is larger in the scenario with more causal variables, but the approaches have all similar errors. The stability of variable selection, i.e. the overlap of selected variables between replicates, is similar across the different methods in the simulation scenario with group size 10 where RFE has a low stability of only 58% compared with values between 76 and 87% for the other methods. Moving to the simulation scenario with group size 50, the stability drops slightly for the top-performing methods such as Boruta and Vita. A more dramatic decrease is shown for RFE, which, as mentioned before, aims at selecting a minimum set of variables. Thus it is expected that the selected variables do not overlap between replicates.

**Figure 2:**
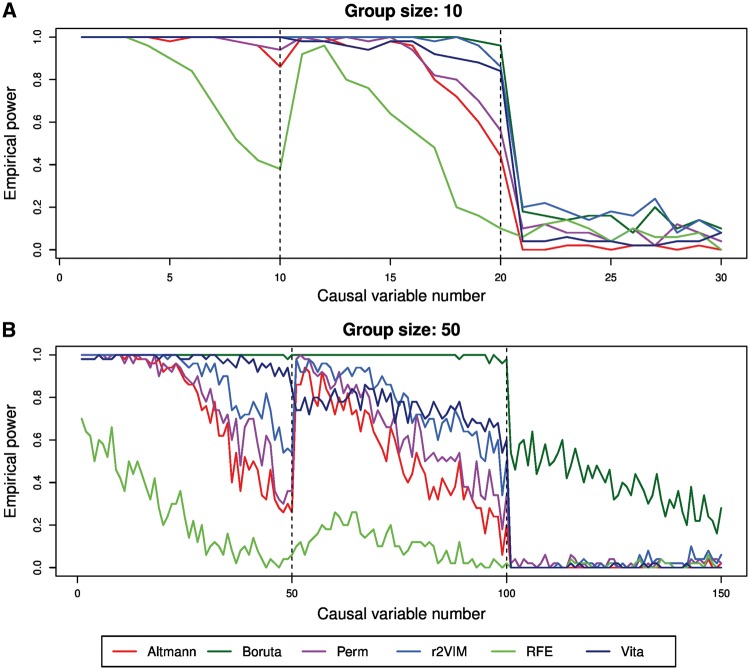
Empirical power to select causal variables in Simulation Study 1. Shown is the empirical power of each causal variable in the simulation scenarios with group sizes of 10 with a total of 30 causal variables (**A**) and 50 with a total of 150 causal variables (**B**). Each of the variable selection approaches is given in a different color.

Sensitivity is a global measure across all causal variables, but it is also of interest to evaluate the power of the different approaches for each causal variable separately. Figure 2 therefore shows the empirical power for each of the 30 and 150 causal variables in the simulation scenarios with group size 10 and 50, respectively.

Overall, empirical power decreases from 100% for the first variables in Group 1 to <10% for variables in Group 3. This finding is consistent with the simulated correlation between variables and outcome since variables in Group 1 and 2 were simulated to have a larger effect on the outcome. In addition, for some methods power decreases within groups, which can be explained by decreasing correlation with the base variable and therefore with the outcome.

Boruta has the largest empirical power across settings and variable groups. In the simulation setting with group size 10, r2VIM is more powerful than Vita, whereas the latter method has more power for the lower correlated variables in Groups 1 and 2 for the setting with group size 50. Interestingly, Vita is the only method for which the first variable in Group 2 has a lower power than the last variable in Group 1. Note that this comparison cannot be performed for the Boruta method because most of the variables in the two groups are detected in all replicates. As expected, RFE is the least powerful method.

**Figure 3:**
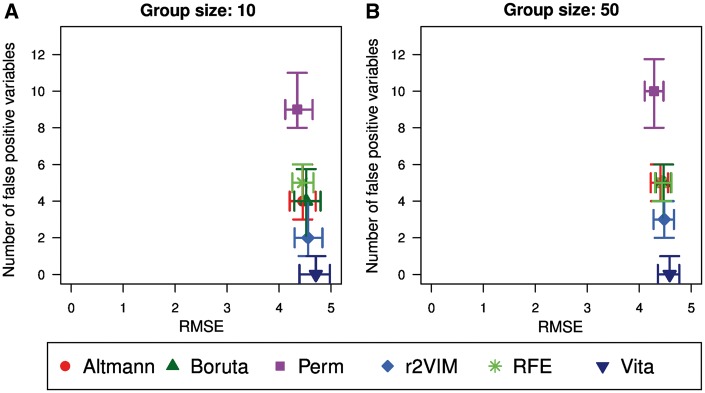
Performance comparison in Simulation Study 1 based on null model. Shown are RMSE versus number of falsely selected variables of the scenarios with outcome simulated independently of any predictor variables using group sizes of 10 (**A**) and 50 (**B**). Each subfigure displays the median as well as the interquartile range over all 50 replicates of each method using different plotting symbols and color.

Results for the two simulated null scenarios where the outcome is independent of any of the predictor variables are shown in Figure 3.

In general, the RMSEs and the numbers of falsely selected variables are similar in the two scenarios with group sizes of 10 and 50. The RMSEs are much larger than in the settings with true effects but they are in the same range for all methods. In contrast, the median number of falsely selected variables ranges from 0 for Vita to 10 for Perm out of the 5000 simulated predictor variables. Boruta detects four to five variables on average.

### Simulation Study 2

**Figure 4:**
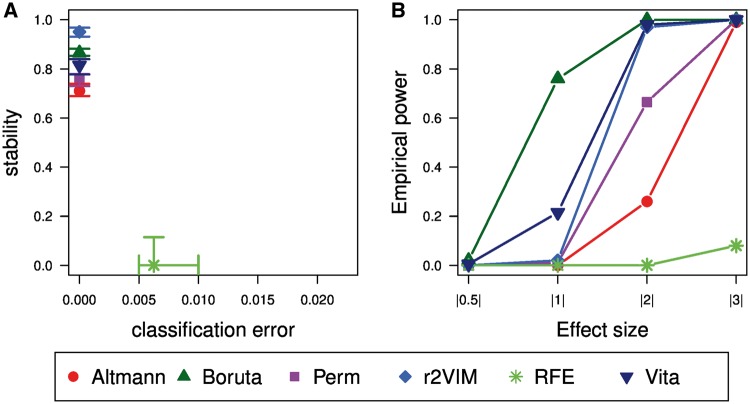
Performance comparison in Simulation Study 2. Shown are classification error versus stability (**A**) and empirical power depending on the absolute effect size (**B**). For classification error and stability the median as well as the interquartile range over all 50 pairs of replicates are displayed. For empirical power the median frequency per category of absolute effect size is given. Results for each method can be distinguished by plotting symbols and colors.

The second simulation study was conducted to compare the performance of the variable selection methods under realistic correlation structures. The results are summarized in Figure 4.

In Figure 4A the parameters classification error and stability are displayed. Again, RFE is different from the other methods. The classification error is low but considerably larger than the classification error of the other methods that achieve a perfect classification. Median stability is zero meaning that different sets of variables are selected in each pair of replicates, which are simulated using the same causal variables and effect sizes. In contrast, the stability of the other methods ranges from around 70% to close to 100%. With a stability of approximately 95%, r2VIM is the most stable approach, followed by Boruta, Vita, Perm and Altmann.

The empirical power as a function of absolute effect size is shown in Figure 4B. Variables with an effect size of 0.5 are never selected by any of the methods. In contrast, variables with the highest effect size of 3 are always selected by all methods except RFE. For variables with moderate effect sizes Boruta outperforms all methods. The second most powerful approach is Vita, which is similar to r2VIM concerning the larger effect size of 2. Empirical power of RFE is generally low and only variables with large effect sizes are selected.

### Experimental data sets

**Figure 5:**
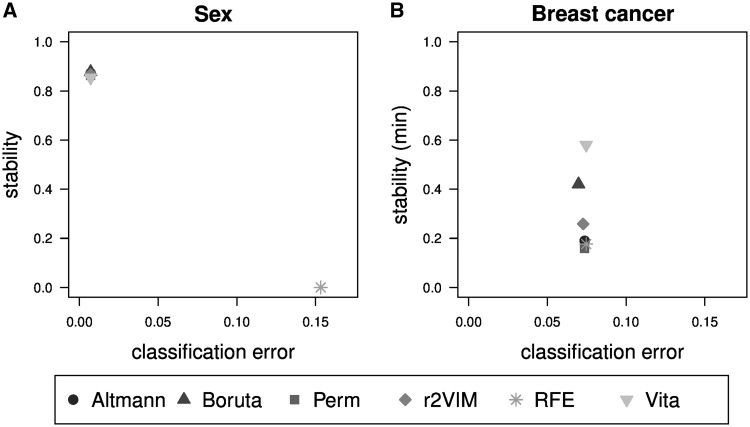
Performance comparison based on experimental data sets. Shown are classification error versus stability of the two experimental studies predicting sex (**A**) and estrogen receptor positive breast cancer (**B**). Each subfigure displays the median error and variable stability of the two different data sets that were analyzed for each research question using different plotting symbols and shades of gray. Note that a different definition of stability is used in subfigure (B), which is defined relative to the minimum and not the union of the two sets of selected variables.

Because the causal predictor variables are unknown in the experimental data sets the different variable selection methods can only be compared based on classification error and stability of variable selection as shown in Figure 5. Stability is determined here by comparing the two available data sets for each condition.

In the sex classification problem, all methods, except RFE, show similar good prediction performances with classification errors <0.01. In addition, the stability of variable selection is approximately 85% for these methods. In contrast, RFE is unstable, owing to high correlation between different CpG positions on the X chromosome. Only two positions in each data set are selected by RFE and because they are different, the resulting stability of this method is zero (Supplementary Table S1).

In the breast cancer problem, the classification error is larger but similar for all methods. Most methods select considerably more of the 12 592 common genes in the RNA-Seq data set compared with the array data set (Supplementary Table S1). Hence, a different definition of stability was used based on the minimum instead of the union of the two sets of selected variables. Based on this criterion, Vita is the most stable method with a stability of 58%, followed by Boruta with 42%. If the original stability definition is used all methods feature stability values <20% (Supplementary Figure S1).

Supplementary Tables S2 and S3 list the selected CpG positions and genes per data set and method. Supplementary Figure S2 shows the overlap between selected variables across the different methods. In the sex classification problem, most of the CpG positions have been identified using several methods but Vita has selected >100 unique variables. And Vita is the only approach that uniquely detected three CpG sites on the autosomes. All other CpG sites are located on the X chromosome and can therefore be considered to be true findings. Prediction of estrogen receptor positive breast cancer results in more diverse sets of selected genes across methods. Only two genes have been identified by every method. One is the gene *ESR1* (estrogen receptor 1), which encodes the estrogen receptor-alpha. The second gene is *AGR3* (Anterior Gradient 3), which has been associated with estrogen receptor status in breast cancer at gene and protein expression levels [45, 46].

**Figure 6:**
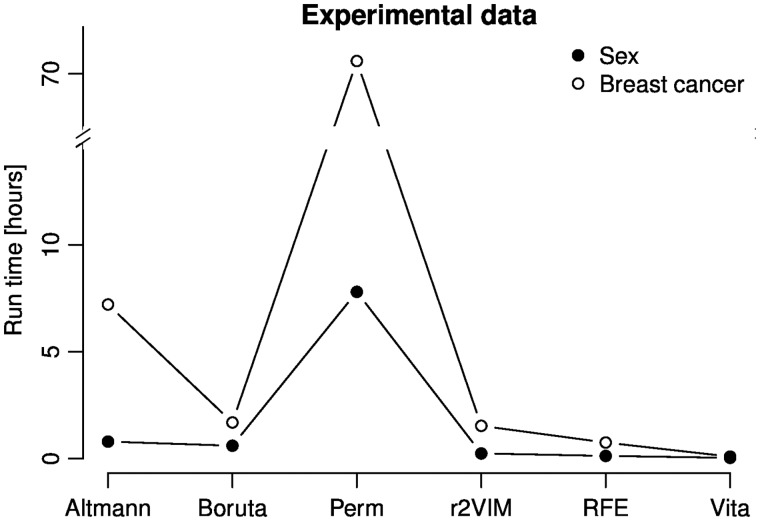
Run time comparison based on the classification of experimental data sets. Shown are run times (in hours) for each method as an average of the two data sets of each research question.

Figure 6 compares the average run time of each method for the two research questions.

As expected, Perm is time-consuming for large omics data sets with >70 h of run time on the breast cancer data sets. Although the analysis of the sex classification problem took only approximately 8 h, Perm was still the slowest approach in our comparison. In contrast, Boruta finished within 2 and 1 h, respectively, while Vita was even more efficient with <6 and 2 min, respectively.

## Discussion and conclusion

We compared different variable selection approaches that were recently proposed to identify all relevant predictor variables within a classification or regression problem. In addition, we included in our comparison a popular method with a different aim, i.e. to find a small subset of important variables with an optimal prediction performance. Both simulation studies identified Boruta as the most powerful approach, followed by the Vita method. Under a pure null model without any predictor variables related to the outcome, Vita was the most robust approach.

Simulation studies were designed so that performance differences between the variable selection methods were observed. In contrast, evaluation criteria of all approaches except RFE were similar in the analysis of the experimental data sets. However, Vita demonstrated slightly better stability in variable selection than Boruta. The large overlap of selected CpG positions in the sex classification example is caused by the strong signal in the data sets because most of the positions on the X chromosome show large methylation differences between females and males. Effects are smaller and more variable in the breast cancer data sets so that results of the different methods are less consistent. For the analysis of large omics data sets we recommend Boruta and Vita, which is considerably faster. Because the different approaches use the same implementation of the RF algorithm, the differences in run time are owing to the number of RFs that are built with each method. For example, the Vita approach generates two forests, whereas the Perm method creates a complete new forest for each permutation. Using Vita, selecting variables and training the final RF model in a data set with 12 000 genes and 500 individuals was finished in <10 min. Thus, this approach will also be feasible for much larger data sets, e.g. measuring more than a million CpG positions in sequencing-based methylation studies, genetic variants genotyped on microarrays or using NGS. However, Vita, similar to r2VIM, estimates the null distribution of variable importance using provided predictor variables only. Therefore, this approach is only applicable in the setting of high-dimensional data sets where the assumption is valid that many variables are not important for predicting the outcome. In contrast, Boruta uses the concept of shadow variables, i.e. permuted versions of the original variables, and can therefore also be applied on the more traditional low-dimensional data with many more individuals than variables.

Both, Boruta and Vita have a single and easily interpretable parameter, which defines a threshold for the internally calculated empirical *P*-values to identify relevant variables. In contrast, the other methods have two arguments, which need to be set by the user or alternatively tuned using e.g. cross-validation procedures. Some of the methods might not be robust regarding those parameter values, which might lead to diverse results (data not shown).

A popular alternative to the RF approach for variable selection is penalized regression methods (also called regularized or shrinkage regression methods) such as Least Absolute Shrinkage and Selection Operator [47] or elastic net [48], which have been applied to omics data sets [49, 50]. The general idea is to add a penalty to the loss function so that regression coefficients are shrunken toward zero resulting in a sparse model. The performance of different types of penalized regression methods has been evaluated in several studies, e.g. [51, 52]; however, to the best of our knowledge, no comprehensive and neutral study comparing RF and penalized regression methods has been performed regarding selection of all relevant variables, which was the focus of the current study. 

As shown previously, the different variable selection methods lead to models with similar prediction performance; however, the sets of selected variables are different [28]. However, agreement and stability of variable selection depend strongly on the effect size of the predictor variables. Large sets of overlapping variables can be found if many predictor variables show strong association with the outcome as in the sex prediction problem based on methylation data.

The results of the simulation studies are similar for both regression and classification settings, and the power of the methods is mainly influenced by the effect sizes of the predictor variables. Both simulation models contained some causal variables with small effects, which were not detected with reasonable power by any of the methods. The small overlap in selected genes in the experimental breast cancer data sets might as well be caused by a power problem. However, stability of variable selection might be improved if external information about biological pathways and networks can be integrated in the model training step [45]. In this situation, stability would not be defined on variable level such as genes but rather on pathways or modules in networks.

In conclusion, we recommend Boruta and Vita for selection of relevant variables in high-dimensional data sets. The Boruta approach can also be applied in low-dimensional settings.

##

**Key Points**

- For interpretation of machine learning prediction methods it is important to identify all relevant variables including those carrying redundant information.
- We performed a systematic evaluation of several variable selection approaches using both simulated and experimental data sets. The Boruta and Vita approaches are sensitive regarding detection of causal variables while controlling the number of false-positive findings at a reasonable level.
- Both methods are available as efficient implementations making analysis of high-dimensional data sets feasible.
- Boruta can also be applied in low-dimensional data sets with more observations than variables.

## Supplementary Material

supplementary_data_new_bbx124

supplementary_tables_2_3_bbx124
