# Supplementary material for: Evaluation of variable selection methods for random forests and omics data sets
Source: Brief Bioinform. 2017 Oct 16;20(2):492–503. doi: 10.1093/bib/bbx124 (PMC6433899; doi:10.1093/bib/bbx124)
Supplement: supplementary_data_new_bbx124 [file supplementary_data_new_bbx124.pdf]

Supplementary material
“Evaluation of variable selection methods for random forests and omics data sets”

Frauke Degenhardt, Stephan Seifert and Silke Szymczak

Contents

1 Supplementary figures **2**

2 Supplementary tables **4**

1 Supplementary figures

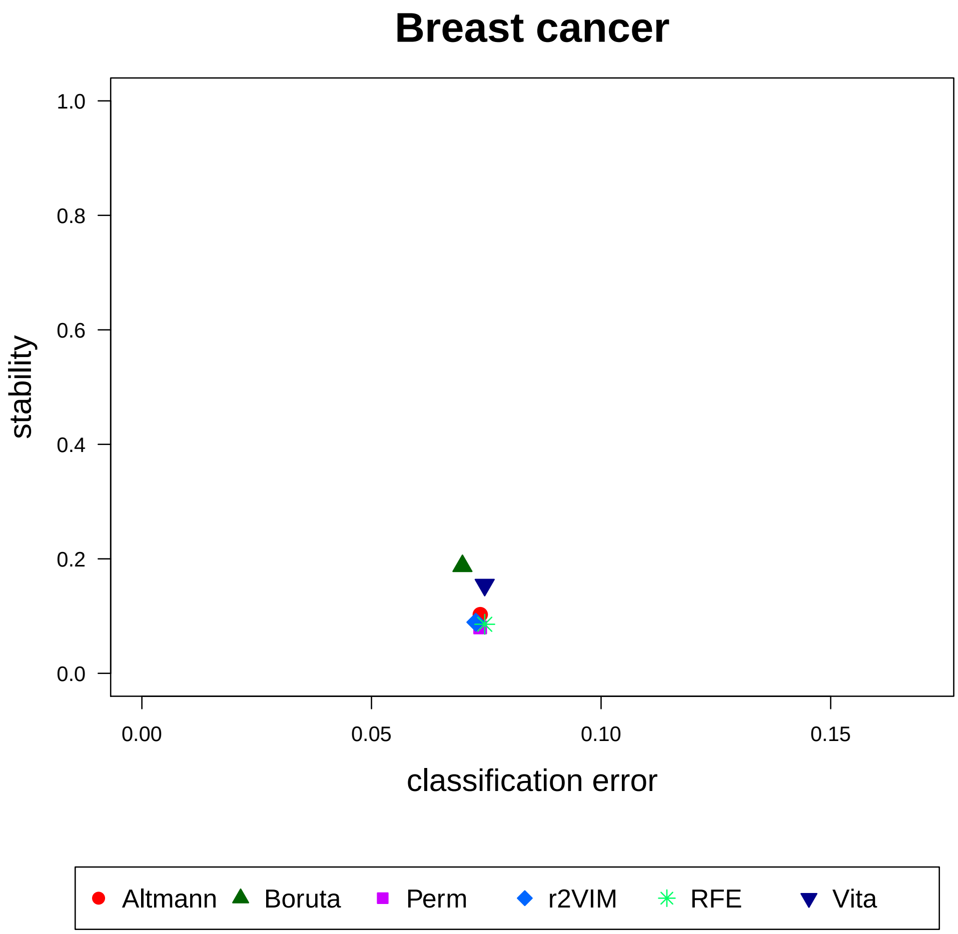

Supplementary Figure 1: Shown are classification error versus stability of predicting estrogen receptor positive breast cancer using the different variable selection approaches. The figure displays the median error and variable stability of the two different data sets that were analyzed using different plotting symbols and color. Note that in contrast to Figure 4 (B) the original definition of stability is used.

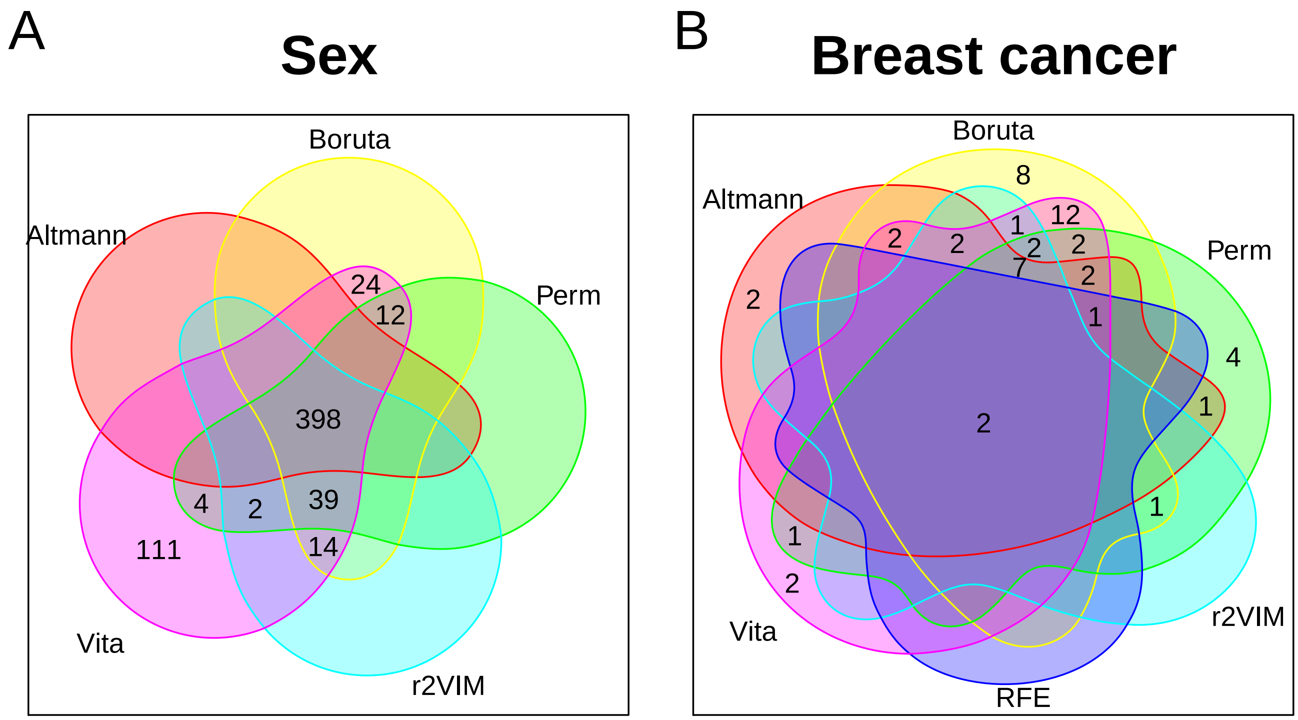

Supplementary Figure 2: Venn diagrams comparing sets of variables commonly identified by each of the variable selection methods on the experimental studies predicting sex (A) and estrogen receptor positive breast cancer (B). For each method only variables identified in both data sets for each research question were used. Note that RFE is not included in subfigure (A) since the two sets of variables were different without any overlaping CpG position.

2 Supplementary tables

research question	data set	Altmann	Boruta	Perm	r2VIM	RFE	Vita
	Adkins	450	542	517	510	2	647
sex	Mozhui	404	500	465	465	2	664
	common	398	487	455	453	0	604
	array	104	100	145	58	17	62
breast cancer	RNA-Seq	100	166	165	125	21	207
	common	19	42	23	15	3	36

Supplementary Table 1: Number of selected variables per experimental data set and method.

Supplementary Tables 2 and 3: additional file `supplementary_tables_2_3.xlsx`
